# The incretin-bone axis: molecular mechanisms and clinical implications

**DOI:** 10.3389/fendo.2026.1907875

**Published:** 2026-10-05

**Authors:** Andrea Muscarà, Federica Bellone, Michela Rossi

**Affiliations:** 1 Department of Clinical and Experimental Medicine, University of Messina, Messina, Italy; 2 Bone Physiopathology Research Unit, Translational Pediatrics and Clinical Genetics Research Division, Bambino Gesù Children’s Hospital, IRCCS, Rome, Italy

**Keywords:** bone cells, fracture risk, GIP, GLP-1, incretin hormones, incretin-based therapy

## Abstract

Incretin hormones, primarily glucose-dependent insulinotropic polypeptide (GIP) and glucagon-like peptide-1 (GLP-1), are key regulators of postprandial glucose homeostasis and exert pleiotropic actions beyond pancreatic β-cell function. Increasing evidence identifies the incretin axis as a critical mediator linking nutrient intake to skeletal remodeling. Preclinical studies demonstrate that incretin signaling influences bone turnover through coordinated effects on osteoblasts, osteoclasts, osteocytes, and mesenchymal stromal cells. These actions involve modulation of major pathways, including cAMP/PKA, MAPK, Wnt/β-catenin signaling, and the RANK-L/OPG axis, ultimately promoting osteogenesis while suppressing bone resorption. Genetic and pharmacological models highlight the importance of intact incretin receptor signaling for maintaining bone mass, microarchitecture, and matrix quality. In particular, GIP appears to exert direct anabolic and antiresorptive effects on bone cells, whereas GLP-1 influences skeletal homeostasis through both direct mechanisms and indirect endocrine pathways. Despite robust mechanistic insights, translation to clinical settings remains challenging. Studies on incretin-based therapies, including GLP-1 receptor agonists and dual GIP/GLP-1 agonists, report heterogeneous effects on bone turnover markers and inconclusive outcomes on fracture risk. A major confounding factor is therapy-induced weight loss, which may negatively impact skeletal integrity and obscure direct drug effects. This review integrates current knowledge on the molecular and cellular mechanisms of incretin action in bone, critically examines available clinical data, and highlights key gaps in the field. A deeper understanding of incretin-bone interactions is essential to clarify whether targeting this axis may represent a viable strategy for preserving skeletal health in metabolic disease.

## 1 Introduction

The regulation of postprandial glucose homeostasis relies on a coordinated interplay between nutrient absorption, neural signaling, and endocrine responses. Incretin hormones represent a central component of this enteroinsular axis. The incretin effect, first recognized in the early 20^th^ century, describes the observation that oral glucose elicits a substantially greater insulin response than an equivalent intravenous glucose load, an enhancement mediated by gut-derived hormones that amplify glucose-stimulated insulin secretion (1, 2). Two peptides fulfill the criteria for incretin hormones in humans: glucose-dependent insulinotropic polypeptide (GIP) and glucagon-like peptide-1 (GLP-1). Both are secreted rapidly after nutrient ingestion and act on pancreatic β-cells to potentiate insulin secretion in a glucose-dependent manner. However, their roles diverge significantly. GLP-1 modulates gastric emptying, suppresses glucagon secretion, reduces appetite signaling and improves cardiovascular function, whereas GIP boosts insulin secretion in a glucose-dependent way stimulates glucagon release mostly during fasting or hypoglycemia and influences adipose tissue metabolism although its effects on cardiorenal system remain controversial (3). Importantly, GLP-1 retains insulinotropic efficacy in individuals with type 2 diabetes mellitus (T2D), whereas GIP responsiveness is often impaired (1, 4).

Emerging evidence positions the incretin axis as a physiological bridge between nutrient intake and bone remodeling. Bone is not only a structural organ but a dynamic, metabolically active tissue responsive to hormonal and nutritional signals. Postprandial incretin release contributes to the acute suppression of bone resorption and to the longer-term modulation of bone formation, a concept now referred to as the gut-bone axis (5–7).

Mechanistically, incretin hormones influence multiple cell types in the bone microenvironment, including osteoblasts, osteoclasts, osteocytes, and mesenchymal stromal cells (MSC), through canonical signaling cascades such as cAMP/PKA, MAPK, and Wnt/β‐catenin, and by altering the RANK-L/Osteoprotegerin (OPG) axis. These pathways converge to promote osteogenesis, restrain resorption and preserve matrix quality, thereby coupling nutrient availability to skeletal maintenance (8, 9).

In addition to canonical kinase signaling, incretin effects on skeletal cells should also be interpreted within the broader framework of epigenetic control of bone remodeling. Osteoblast lineage commitment and maturation are regulated by DNA methylation, histone modifications, chromatin remodeling, and non-coding RNA, which dynamically shape accessibility of osteogenic transcriptional programs including Runx2- and Wnt/β-catenin-dependent gene expression (10, 11). This level of regulation is mechanistically relevant because key pathways already implicated in incretin action, including Wnt/β-catenin and RANK-L/OPG, are themselves epigenetically regulated, suggesting that incretins may influence bone remodeling not only through acute second-messenger cascades but also through transcriptionally stable changes in cell fate and coupling signals (11, 12). Although direct evidence linking GIP or GLP-1 signaling to chromatin remodeling in bone remains limited, recent work in stem and osteoblastic models supports non-coding RNA-dependent and differentiation-stage-specific regulation as a plausible mechanistic layer that deserves consideration in the incretin-bone axis (11, 13).

Preclinical models have provided compelling insights; genetic ablation of incretin receptors or pharmacological modulation of the axis produces marked effects on bone mass, microarchitecture, and tissue material properties. For instance, loss of GIP receptor (GIPr) signaling is associated with reduced bone formation, defective collagen maturation, and increased skeletal fragility, whereas activation of incretin pathways in models of metabolic bone disease can restore aspects of bone quality independently of insulin secretion (14). GLP‐1 receptor (GLP-1r) signaling has been implicated in the regulation of bone resorption both directly, through effects on osteoblast-mediated coupling signals, and indirectly, via stimulation of calcitonin secretion from thyroid C-cells.

Translating these mechanistic findings to the clinic has proven challenging. Human studies report heterogeneous results: some trials and meta-analyses report favorable changes in bone turnover markers with GLP‐1 receptor agonists (GLP-1RA), while large cohort studies have not consistently demonstrated reductions in fracture incidence (15, 16). A major confounder is the pronounced body weight loss induced by many incretin‐based therapies; reduced mechanical loading and altered nutritional status can offset direct anabolic or anti-resorptive effects on bone. Moreover, differences in drug pharmacokinetics, receptor selectivity, and dosing regimens complicate cross‐study comparisons and the extrapolation of animal data to human outcomes.

Given the therapeutic prominence of incretin‐based agents and the high clinical burden of osteoporosis and fracture in populations with metabolic disease, a clearer understanding of how incretin signaling affects bone at molecular, cellular, and whole‐organ levels is urgently needed.

This review provides an extended synthesis of the molecular mechanisms regulating incretin synthesis and secretion, their biological actions on bone remodeling, and their relevance for new therapeutic approaches. By integrating mechanistic insights with clinical observations, we aim to delineate existing knowledge gaps and propose research priorities that will clarify whether and how incretin modulation can be harnessed to preserve skeletal health without compromising metabolic benefits.

## 2 Incretin peptides

### 2.1 Glucagon-like peptide-1

GLP-1 is generated through post-translational processing of the proglucagon gene product, a 160-amino acid precursor encoded by the proglucagon gene located on chromosome 2 (17). The gene is expressed in pancreatic α-cells, enteroendocrine L-cells, and specific brainstem and hypothalamus neurons. Despite the uniformity of the transcript, tissue-specific post-translational processing yields distinct peptide products. In intestinal L-cells, the action of prohormone convertase 1/3 (PC1/3) cleaves proglucagon to equimolar quantities of GLP-1 and GLP-2, together with glicentin, oxyntomodulin, glucagon (18). In pancreatic α-cells, prohormone convertase 2 (PC2) drives cleavage toward glucagon, the major biologically active product in that compartment (1).

Two bioactive forms of GLP-1 are produced in the intestine, GLP-1(7-36)NH_2_ and GLP-1(7-37), with the amidated form representing the predominant circulating isoform (18). L-cells are open-type endocrine cells characterized by marked polarity, with secretory granules localized at the basolateral domain enabling rapid hormone release into portal and systemic circulation in response to luminal nutrients, neural inputs, and paracrine hormonal signals (17). GLP-1 is further thought to exert part of its extra-pancreatic effects through the autonomic nervous system, particularly via vagal afferents targeting hypothalamic and brainstem nuclei (19).

GLP-1 mediates its biological actions by binding to the GLP-1r, a G protein-coupled receptor encoded by the GLP1R gene on chromosome 6p21 (20). The GLP-1r is expressed in multiple tissues, including the endocrine pancreas, gastrointestinal tract, lung, heart, kidney, and several regions of the central nervous system (4). Its primary function is glucose-dependent stimulation of insulin secretion; additionally, it slows gastric emptying, suppresses glucagon release, reduces appetite and confers cardioprotective effects (20).

The presence of GLP-1r in bone cells has long been debated (21, 22). A major source of complexity in the field is the tissue-specific and context-dependent biology of incretin receptors. This uncertainty has been attributed to the use of poorly characterized cell lines, inappropriate primer design, and non-specific antibodies against GLP-1r, particularly in murine models (21). More recent evidence by Pereira et al. supports the expression of GLP-1r in skeletal tissues (23). However Experimental evidence indicates that receptor expression is not static: in osteoblastic MC3T3-E1 cells, both GLP-1r and GIPr increase under high-glucose conditions and during BMP-2-induced differentiation, supporting the concept that receptor availability may vary with metabolic milieu and cell state, highlighting the need for further investigation. The existence of an alternative structurally different GLP-1-responsive receptor, using inositolphosphoglycan instead of cAMP, has been reported in liver, skeletal muscle and osteoblasts, adding further complexity to the receptor pharmacology in bone (21). This controversy is best explained by the combination of condition-dependent expression and methodological limitations rather than a simple presence/absence division. At the mRNA and protein level, GLP1r has been reported as undetectable in murine calvarial MC3T3-E1 pre-osteoblasts and in primary murine osteoblasts using RT-PCR (Real-Time PCR) and Western blot with commercial antibodies (24) and as absent in the pre-osteocytic cell line MLO-A5, while being detected in the late osteocytic line IDG-SW3 and in primary murine osteoblasts and osteoclasts, with expression declining progressively as osteoblasts differentiate toward maturity (25). Conversely, other groups have detected GLP1r mRNA and protein in MC3T3-E1 cells by RT-PCR and Western blot, reporting that expression is modulated by glucose concentration and differentiation stage (26), raising the possibility that discrepant results partly reflect differences in culture conditions, passage number, and differentiation state rather than a fixed cell-autonomous receptor status. An additional layer of complexity was proposed by Nuche-Berenguer et al., who described GLP-1 binding and functional signaling in MC3T3-E1 cells that lack the canonical pancreatic GLP-1r, suggesting the existence of an alternative, non-cAMP-linked receptor for GLP-1 in osteoblastic cells (27). Beyond biological variability, a major technical confound is antibody specificity: numerous commercially available anti-GLP-1r antibodies, including widely used polyclonal reagents, have been shown to generate immunoreactive signal in GLP-1r-*knock*-*out* tissue, indicating non-specific binding rather than true receptor detection (28, 29). Only a small number of monoclonal antibodies have been rigorously validated against *knock*-*out* controls and *in situ* ligand-binding assays in extra-skeletal tissues (30), and equivalent validation in bone-derived cells remains largely lacking; most positive reports of GLP-1r protein expression in osteoblasts and osteoclasts to date have relied on antibodies that have not undergone this level of validation. Receptor density represents a further limitation: even where GLP1r mRNA is detected in bone cells, absolute transcript and protein levels are substantially lower than in classical target tissues such as pancreatic β-cells, placing expression close to the detection limit of standard RT-PCR and immunohistochemistry protocols and increasing the risk of both false-negative and false-positive results depending on assay sensitivity and cycle threshold settings. Taken together, these observations indicate that apparently conflicting reports on GLP-1r expression in bone cannot be resolved without harmonized, antibody-validated protocols applied across defined differentiation stages and cell sources.

Beyond receptor detection issues, GLP-1r expression itself is subject to post-transcriptional control: in MC3T3-E1 pre-osteoblasts, miR-27a-3p directly targets GLP-1r and suppresses downstream AMPK signaling, thereby impairing osteoblast differentiation and autophagy while promoting an inflammatory phenotype (31), indicating that epigenetic regulation of receptor availability may itself contribute to the inconsistent detection of GLP-1r across cell models. A further layer of complexity arises from biased agonism: class B GPCR such as GIPr and GLP-1r can preferentially engage Gαs/cAMP versus β-arrestin-mediated signaling depending on the ligand, with distinct consequences for receptor desensitization and downstream response (32). This has prompted the deliberate development of “unbiased” long-acting GIP analogs for bone-specific studies, selected on the basis of balanced cAMP accumulation, β-arrestin recruitment and internalization profiles (33), underscoring that the skeletal phenotype elicited by different incretin-based ligands may depend on their specific signaling bias rather than receptor occupancy alone.

Insights into the role of GLP-1 in skeletal physiology have largely derived from GLP-1r knock-out mouse models (34). At 10 weeks of age, GLP-1r-deficient mice (GLP-1r KO) exhibited a slight reduction in tibial trabecular bone volume, accompanied by increased osteoclast numbers and enlarged eroded surfaces, without changes in mineral apposition rate and bone formation. At 16 weeks, GLP-1r KO mice exhibited reduced bone strength, as assessed by three-point bending tests, along with decreased cortical thickness and altered cortical microarchitecture (35). Additionally, bone tissue material properties were impaired, characterized by reduced collagen maturity despite a normal degree of mineralization. These findings indicate that a functional GLP-1r is essential not only for the regulation of bone resorption but also for maintaining bone matrix quality.

Notably, GLP-1 was unable to directly modulate osteoclast differentiation or resorptive activity *in vitro*, indicating that its effects on bone resorption are likely indirect (34). Indeed, GLP-1r-deficient animals displayed reduced calcitonin gene expression. It is now established that, in rodents GLP-1r is expressed in thyroid C-cells, where it stimulates calcitonin secretion (36).

### 2.2 Glucose-dependent insulinotropic polypeptide synthesis and gene regulation

GIP is a 42-amino-acid peptide synthesized and secreted by enteroendocrine K-cells, which are predominantly located in the proximal segment of the small intestine (37). These cells are polarized, with GIP-containing secretory granules mainly distributed at the basal pole to facilitate their basolateral release. According to their morphological and functional characteristics, GIP secretion from K-cells is regulated by neural inputs, hormonal signals, and intraluminal nutrients. GIP circulating levels are low in the fasted state and rise within minutes of food ingestion (4); the hormone is also expressed in specific neuronal populations of the central nervous system, where it has been implicated in neuronal survival and behavioral regulation.

In humans, proGIP is a 153-amino acid polypeptide encoded by six-exon gene on chromosome 17q. Prohormone convertase 1/3 mediates cleavage at single arginine residues to yield the biologically active GIP1–42 fragment, primarily derived from exons 3 and 4. Like GLP-1, GIP contains an alanine at position 2, making it an excellent substrate for Dipeptidyl peptidase-4 (DPP-4) and resulting in rapid conversion to the inactive metabolite GIP(3-42). Intraluminal nutrients not only stimulate GIP secretion but also upregulate GIP gene transcription, establishing a feedforward relationship between luminal nutrient availability and hormone production. GIP signals through GIPr, a class B G protein-coupled receptor encoded by a gene of 14 exons spanning approximately 14.2 kb and is located on chromosome 19q13.3 (38). GIPr expression has been documented in endocrine pancreas, gastrointestinal tract, adipose tissue, adrenal cortex, pituitary gland, vascular endothelium, and several regions of the central nervous system and in bone cells, where mRNA and protein expression have been confirmed in osteoblasts and osteoclasts (39, 40). In osteoblasts, GIPr activation increases intracellular cAMP and calcium, promoting the expression of type I collagen, alkaline phosphatase (ALP) lysyl oxidase, and enzymatic collagen cross-linking (41). GIP attenuated resorptive activity of mature osteoclasts, though the precise molecular mechanisms remain under investigation (42).

The physiological function of the GIP-bone axis has been established through genetically engineered mouse models. Two independent GIPr knock-out mouse models have been described, involving deletion of exons 4–5 or exons 1-6 (43, 44). These regions encode portions of the extracellular domain involved in ligand binding (exons 4-5) or the entire extracellular domain together with the first transmembrane segment (exons 1-6). Despite displaying divergent trabecular bone phenotypes, attributable to differences in transgenic strategy, both models indicate that the absence of a functional GIPr severely impaired both trabecular and cortical bone compartments. Interpretation of these murine models requires caution, as GIP shows considerably greater cross-species sequence variation than GLP-1, which is virtually identical across mice, rats and humans; rat GIP differs from human GIP at two amino acid positions that cause the two ligands to act differently on the rat GIPr (33). This divergence extends to receptor pharmacodynamics: the human GIP receptor is substantially more prone to internalization than its rodent orthologues, with the mouse GIPr in particular showing reduced desensitization, internalization and β-arrestin recruitment relative to the human receptor, an effect that becomes more pronounced with long-acting GIP analogs (45). These species-dependent properties may partly explain why skeletal phenotypes observed in murine GIPr models do not always translate proportionally to human data. Moreover, these models exhibit alterations in bone tissue material properties, including reduced mineralization levels and decreased collagen maturity within the bone matrix. Collectively, these changes in bone mass, microarchitecture, and material properties contribute to increased skeletal fragility. Notably, loss of GIPr function has also been associated with compensatory upregulation of GLP-1 sensitivity, complicating the attribution of phenotypic features to GIP signaling *per se*.

To determine whether the skeletal alterations observed in GIPr knock-out mice were directly attributable to the absence of GIP signaling or to compensatory mechanisms related to increased GLP-1 sensitivity, the bone phenotype of double incretin receptor knockout (DIRKO) mice, lacking both GIPr and GLP-1r, was investigated (35). These animals similarly exhibited marked reductions in bone mass, along with significant alterations in trabecular and cortical microarchitecture and tissue material properties, thereby underscoring the critical role of incretin signaling in bone homeostasis (35). Additional evidence linking GIP to human bone metabolism comes from a cohort of peri-menopausal women carrying the rs1800437 single-nucleotide polymorphisms, associated with reduced GIPr activity, who exhibited lower femoral neck and total hip bone mineral density and a higher incidence of non-vertebral fractures (46).

## 3 Role of incretins in bone remodeling

The skeletal effects of incretin hormones are mediated through a complex and highly integrated network of cellular interactions involving osteoblasts, osteoclasts, osteocytes, and mesenchymal stem/stromal cells. Rather than acting through a single linear pathway, incretins orchestrate bone remodeling by modulating multiple signaling cascades and intercellular communication systems, ultimately coupling nutrient availability to skeletal homeostasis. Evidence from *in vitro* systems, animal models, and human studies collectively supports a model in which incretin signaling exerts predominantly anabolic and anti-resorptive effects on the skeleton, although these responses are context-dependent and influenced by metabolic status, hormone concentrations and the specific receptor engaged (**Figure 1**). Incretins modulate several key pathways involved in the bone cells network (**Figure 2**), including:

**Figure 1:**
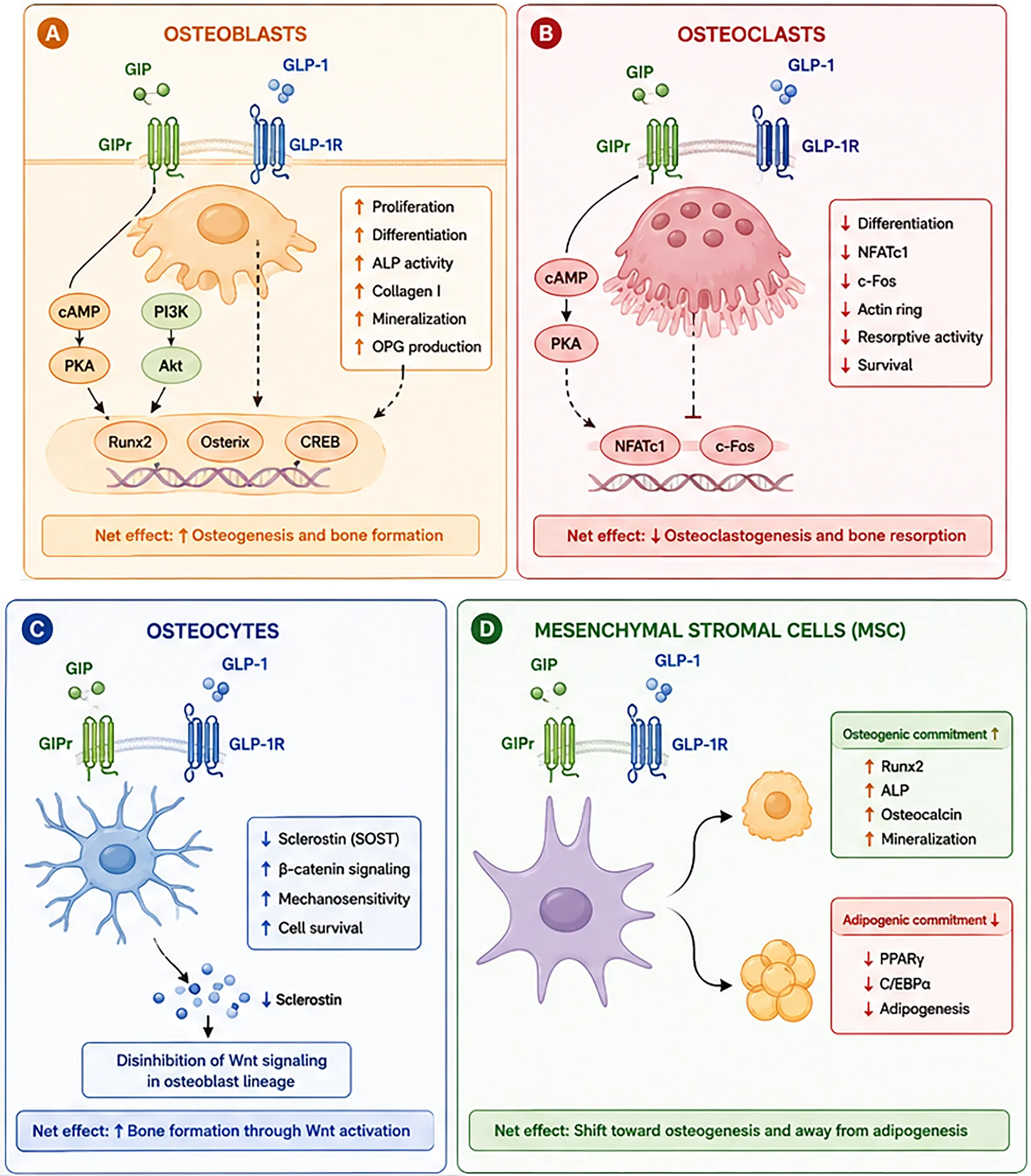
Effects of incretin hormones on bone cells. Graphical representation of the effect of GIP and GLP-1 on **(A)** Osteoblasts, **(B)** Osteoclasts, **(C)** Osteocytes and **(D)** Mesenchymal Stromal Cells (MSC). Cell-type-specific mechanisms of incretin action in bone. GIP and GLP-1 engage their cognate receptors (GIPr and GLP-1r) on osteoblasts, osteoclasts, osteocytes and mesenchymal stromal cells (MSC), triggering distinct downstream signaling cascades and net skeletal effects in each lineage. **(A)** Osteoblasts. GIPr/GLP-1r activation increases intracellular cAMP, activating protein kinase A (PKA), and stimulates PI3K/Akt signaling. These pathways converge on the transcription factors Runx2, Osterix and CREB, driving osteoblast proliferation, differentiation, alkaline phosphatase (ALP) activity, type I collagen synthesis, matrix mineralization, and osteoprotegerin (OPG) production. Net effect: increased osteogenesis and bone formation. **(B)** Osteoclasts. GIPr/GLP-1r signaling on osteoclast precursors activates cAMP/PKA, which suppresses the pro-osteoclastogenic transcription factors NFATc1 and c-Fos. This results in reduced osteoclast differentiation, disruption of the actin ring required for resorption, decreased resorptive activity, and reduced osteoclast survival. Net effect: decreased osteoclastogenesis and bone resorption. **(C)** Osteocytes. GIPr/GLP-1r activation reduces expression of sclerostin (SOST), while increasing β-catenin signaling, mechanosensitivity, and osteocyte survival. The resulting decrease in secreted sclerostin relieves its tonic inhibition of Wnt signaling in the osteoblast lineage. Net effect: increased bone formation through disinhibition of Wnt/β-catenin activation. **(D)** Mesenchymal stromal cells (MSC). GIPr/GLP-1r signaling shifts MSC lineage commitment toward osteogenesis, marked by increased Runx2, ALP, osteocalcin, and mineralization, while concurrently suppressing adipogenic commitment, evidenced by decreased PPARγ, C/EBPα, and adipogenesis. Net effect: a coordinated shift toward osteogenesis and away from marrow adipogenesis. Abbreviations: ALP, alkaline phosphatase; Akt, protein kinase B; cAMP, cyclic adenosine monophosphate; CREB, cAMP response element-binding protein; C/EBPα, CCAAT/enhancer-binding protein alpha; GIP, glucose-dependent insulinotropic polypeptide; GIPr, GIP receptor; GLP-1, glucagon-like peptide-1; GLP-1r, GLP-1 receptor; NFATc1, nuclear factor of activated T-cells 1; OPG, osteoprotegerin; PI3K, phosphoinositide 3-kinase; PKA, protein kinase A; PPARγ, peroxisome proliferator-activated receptor gamma; Runx2, runt-related transcription factor 2; SOST, sclerostin gene/protein.

**Figure 2:**
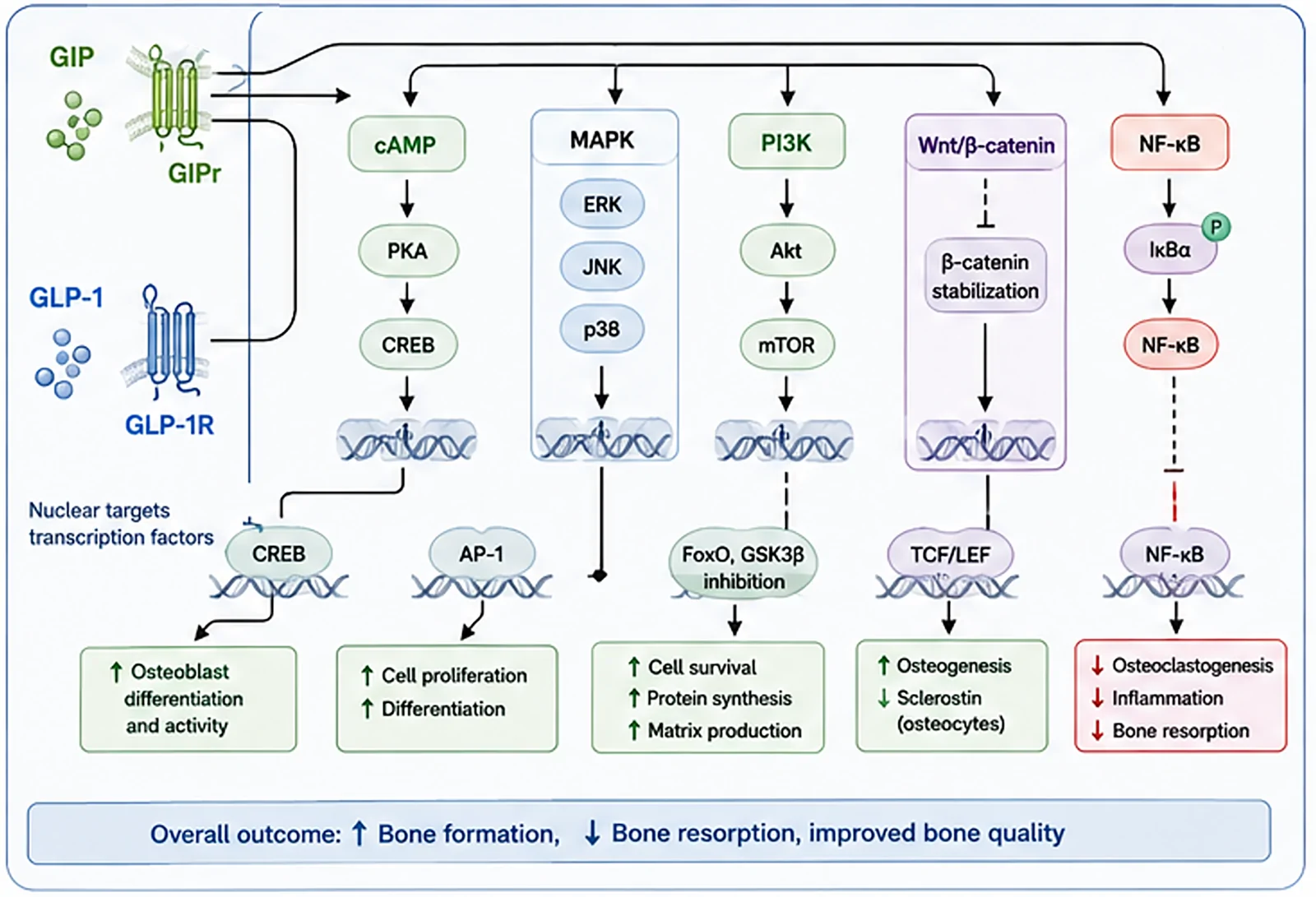
Molecular signaling pathways activated by GIP and GLP-1 in bone cells.

• cAMP/PKA signaling, regulating osteoblast differentiation, matrix synthesis, and survival;
• MAPK pathways, controlling osteoblast and MSC proliferation, differentiation and migration;
• Wnt/β-catenin signaling, promoting osteogenesis and inhibiting marrow adipogenesis;
• RANK-L/OPG axis, modulating the differentiation and activity of osteoclasts;
• NF-κB and NFATc1 pathways, regulating osteoclastogenesis.

Through simultaneous modulation of these pathways across cell types, incretins promote a coordinated shift toward bone formation while suppressing resorption. This integrated response is particularly evident in the postprandial state, where rapid incretin release acts as a physiological coupling signal between nutrient intake and skeletal metabolism.

### 3.1 Effects of incretins on osteoclasts: suppression of bone resorption and functional modulation

Incretin hormones exert a complementary anti-resorptive effect by targeting osteoclast differentiation and activity. Both direct and indirect mechanisms are involved in this regulation. Direct anti-resorptive effects are mediated primarily through GIPr signaling. Experimental studies have demonstrated that GIP inhibits osteoclastogenesis by interfering with RANK-L-induced differentiation pathways, downregulating key transcription factors such as NFATc1 and c-Fos, and suppressing NF-κB and MAPK cascades, reducing osteoclast maturation and resorptive capacity (47). In addition to inhibiting differentiation, GIP impairs osteoclast function by disrupting cytoskeletal organization, particularly the formation of the actin ring structure required for bone resorption, leading to decreased resorptive activity at the bone surface (42). Murine models further support these findings, indeed GIPR-deficient mice exhibit increased osteoclast numbers and activity, contributing to an imbalance favoring bone resorption (42, 48). Conversely, pharmacological activation of incretin signaling in animal models, including ovariectomized mice, reduces osteoclast surface and bone resorption markers, thereby mitigating bone loss (49). The estrogen-deficient state itself appears to modify how GLP-1r agonism engages the skeleton: a 2025 study in ovariectomized mice showed that exendin-4 improved bone strength, trabecular volume and collagen maturity, but that these effects were preserved only when the compound could access the central nervous system, being lost with a brain-restricted GLP-1r agonist despite intact calcitonin stimulation (50). This suggests that, in estrogen deficiency, the skeletal benefit of GLP-1 depends on a central relay rather than on direct peripheral receptor engagement, a mechanistic nuance not captured by studies conducted in gonadally intact animals. These data indicate that incretin signaling is essential for restraining osteoclast-mediated bone degradation under both physiological and pathological conditions.

Incretin signaling also interacts with other hormonal systems that are central to skeletal homeostasis. GLP-1 stimulates calcitonin secretion from thyroid C-cells in rodents, providing an additional endocrine axis through which GLP-1r signaling suppresses osteoclast activity (36). The relevance of this axis in humans is less certain because thyroid incretin receptor expression differs substantially across species; however, human studies have demonstrated that incretin release following nutrient intake is associated with rapid suppression of circulating bone resorption markers such as β-C-terminal telopeptide of type I collagen (β-CTX). This postprandial anti-resorptive response underscores the physiological relevance of incretin-mediated regulation of osteoclast function.

Regarding GIP, a randomized crossover study in patients with hypoparathyroidism demonstrated that meal- and GIP-induced suppression of bone resorption markers was preserved despite the absence of PTH secretion, whereas the GLP-2-induced effect was not, indicating that GIP’s antiresorptive action does not require an intact PTH axis (51). This is consistent with acute infusion studies in patients with type 1 diabetes (T1D), in which GIP robustly reduced bone resorption independently of insulin secretion and glycemic level, with only a modest suppressive effect on PTH observed under hyperglycemia (52). Together, these data position PTH as a modulator rather than an obligatory mediator of GIP-driven bone resorption suppression.

### 3.2 Effects of incretins on mesenchymal stromal cells: regulation of lineage commitment

MSC represent a critical upstream target of incretin action, as they give rise to osteoblasts, adipocytes, and other mesenchymal lineages within the bone marrow niche (53). The balance between osteogenic and adipogenic differentiation of MSC is a key determinant of bone mass and incretin signaling shifts this balance toward osteoblastogenesis. Both GIP and GLP-1 have been shown to promote osteogenic differentiation of MSC while inhibiting adipogenesis, through activation of MAPK and Wnt/β-catenin pathways and upregulation of Runx2 (54). This action counteracts the age-related and metabolic drift toward increased marrow adiposity, a phenomenon associated with both osteoporosis and insulin-resistant states. Pharmacological activation of incretin pathways in murine systems expands osteoblast progenitor populations and enhances bone formation, while impaired incretin signaling reduces osteogenic potential and increased marrow fat accumulation (55). The regulation of MSC lineage commitment by incretins represents a potentially important upstream mechanisms through which these hormones maintain the regenerative capacity of the skeletal system, particularly in the context of aging and metabolic disease (56). However, human data addressing incretin effects on MSC biology and the osteogenic-adipogenic balance in bone marrow are scarce and represent a critical knowledge gap.

### 3.3 Effects of incretins on osteoblasts: induction of osteogenesis

Osteoblasts represent the primary target of incretin-mediated anabolic signaling in bone. Both GIP and GLP-1 directly modulate osteoblast differentiation, activity, and survival through receptor-mediated mechanisms (53).

At the molecular level, activation of GIPr and GLP-1r activation in osteoblastic cells leads to increased intracellular cyclic AMP (cAMP) levels and activates downstream protein kinase A (PKA), which drives the transcriptional programs essential for osteogenesis. This cAMP/PKA signaling axis promotes the expression of canonical osteogenic markers, including ALP, type I collagen, osteocalcin, and the fundamental transcription factors Runx2 and Osterix. At the same time, incretin hormones activate MAPK and PI3K/Akt, which contribute to osteoblast proliferation, differentiation, and resistance to apoptosis.

These observations are consistently supported by studies in osteoblast-like cell models, such as MC3T3-E1, MG-63, and Saos-2 cells, in which incretin exposure enhances mineralized nodule formation, collagen synthesis, and overall osteoblastic function (57). Incretins also modulate cytoskeletal organization and promote osteoblast migration, a critical step in the recruitment of bone-forming cells to resorption sites; this process involves activation of p38 MAPK and RhoA signaling, which modulate actin dynamics and cytoskeletal reorganization.

Importantly, evidence from murine models has provided strong *in vivo* validation of these observations. Mice lacking the GIPr displayed reduced bone formation, decreased bone mineral density (BMD), and impaired microarchitectural integrity associated with diminished osteoblast activity and defective bone matrix deposition, indicating that GIP signaling is essential for normal osteoblast function (42). Conversely, transgenic models with enhanced GIP signaling display increased bone formation markers and improved bone mass, further supporting a direct anabolic role of incretins on osteoblasts. In addition to their direct effects, incretins influence osteoblast-mediated coupling signals that regulate osteoclastogenesis. Specifically, GLP-1 signaling has been shown to increase OPG expression while reducing RANK-L, thereby shifting the balance toward inhibition of osteoclast differentiation (55). This highlights the central role of osteoblasts as intermediaries through which incretins coordinate both arms of the remodeling cycle.

### 3.4 Effects of incretins on osteocytes: regulation of mechanotransduction and WNT signaling

Osteocytes, the most abundant and long-lived cells in bone tissue, are embedded within the mineralized matrix and form an extensive lacuna-canalicular network that coordinates skeletal adaptation to both mechanical and endocrine stimuli. Beyond their traditional role as terminal differentiated osteoblasts, osteocytes serve as key regulators of bone remodeling by integrating mechanical and biochemical signals (58). Although the effects of incretin hormones on osteocytes are less characterized compared to those on osteoblasts and osteoclasts, preliminary preclinical evidence suggests a possible interaction with osteocyte-derived Wnt regulators (59). One primary mechanism involves the regulation of sclerostin, a glycoprotein produced by osteocytes that is a potent antagonist of canonical Wnt/β-catenin signaling and suppresses osteoblast activity (60). In particular, one experimental study reported that exendin-4 reduced SOST/sclerostin mRNA and protein expression in osteocyte-like MLO-Y4 cells and lowered circulating sclerostin levels in diabetic rats (61). However, direct evidence that GLP-1 or GIP regulates sclerostin transcription or secretion in osteocytes through receptor-mediated mechanisms remains limited, and corresponding evidence in humans is currently lacking (59, 62). Therefore, the osteocyte-sclerostin axis should be regarded as a plausible but still insufficiently validated component of incretin action on bone.

The relevance of osteocyte-mediated Wnt regulation is further supported by genetic models of altered incretin signaling, suggesting that incretins influence osteocyte biology beyond indirect effects on surface cells (63). In both GLP-1r-deficient and DIRKO mice, skeletal abnormalities extend beyond alterations in osteoblast and osteoclast activity and include impaired bone microarchitecture, reduced cortical thickness, and deterioration of bone material properties. Histomorphometric analyses have additionally reported changes in osteocyte lacunar characteristics and altered expression of osteocyte-derived regulatory proteins, suggesting that osteocytes may participate directly in the skeletal phenotype associated with defective incretin signaling. Similar observations have been described in GIP receptor-deficient mice, which exhibit impaired matrix quality and increased skeletal fragility despite relatively modest changes in bone turnover indices, pointing to a potential role of osteocytes in the regulation of matrix maturation and mineral organization (14, 34, 35).

A second emerging area concerns mechanotransduction. Osteocytes are the primary mechanosensory cells of bone and translate fluid shear stress and mechanical loading into biochemical signals that regulate remodeling (64). This process involves a highly coordinated network including integrins, connexin-43 gap junctions, primary cilia, and mechanosensitive ion channels such as Piezo1. Mechanical loading normally suppresses sclerostin production and activates Wnt signaling, whereas unloading and immobilization induce osteocytic sclerostin expression and promote bone loss. Although direct evidence remains limited, incretin signaling appears to interact with these mechanotransduction pathways. Experimental administration of GLP-1RA has been associated with preservation of bone microarchitecture and improvements in bone strength disproportionate to changes in bone mass alone, raising the possibility that incretins modulate osteocyte responsiveness to mechanical stimuli and contribute to maintenance of the lacuna-canalicular network (23, 50). This interaction may be particularly relevant in the clinical setting of incretin-based weight-loss therapies. Reduction in body weight decreases skeletal loading, a stimulus normally sensed by osteocytes. Consequently, osteocytes may represent a key site where the potentially beneficial direct skeletal actions of incretins intersect with the adverse consequences of reduced mechanical loading. This area remains insufficiently explored and warrants further investigation, particularly given the role of osteocytes a sensor of the mechanical consequences of weight loss.

In addition to sclerostin regulation, osteocytes may contribute to incretin-mediated remodeling through modulation of osteocyte-derived RANK-L and FGF-23 production. Given that osteocytes constitute a major source of RANK-L in adult bone, changes in osteocyte signaling could influence osteoclastogenesis independently of osteoblastic pathways. The potential interaction between incretins and osteocyte-derived phosphate-regulating hormones remains largely unexplored. These observations collectively support the concept that osteocytes are not passive targets but active mediators of the incretin-bone axis.

Overall, current evidence places osteocytes at the intersection of nutrient sensing, mechanotransduction, and Wnt signaling. However, there are really few direct studies investigating GLP-1r and GIPr expression, downstream signaling pathways, and functional responses in primary osteocytes. Future research should prioritize osteocyte-specific genetic models and mechanistic studies addressing the effects of incretin signaling on sclerostin production, lacuno-canalicular network integrity, and mechanosensory function.

## 4 Clinical evidence: incretin-based therapies

Despite the well-established cardio-reno-hepato-metabolic benefits of GLP-1RA (65), their role in bone remodeling remains uncertain, with clinical evidence still inconclusive. Available studies assessing bone turnover markers are limited in number and yield heterogeneous findings, often confounded by relevant clinical factors, especially the rapid weight loss associated with these therapies, which may adversely affect skeletal biomechanics and contribute to bone loss. This issue is particularly relevant because GLP-1RA-induced weight reduction (66, 67), while metabolically advantageous, is linked to decreased bone mass (68). In addition, the anorexigenic effects of these agents may further limit their potential application in osteoporosis management, as adequate nutritional intake is essential for skeletal maintenance. Consequently, clarifying the direct, weight-independent effects of GLP-1 receptor activation on bone metabolism remains a central challenge.

In this context, Alalwani et al. in a recent meta-analysis of 33 randomized controlled trials (RCT) with bone-related outcomes demonstrated that GLP-1RA were associated with a significant reduction in bone resorption, as reflected by decreased β-CTX levels [SMD (Standardized Mean Differences) -0.36; 95% CI: -0.53, -0.20], a concomitant increase in bone formation markers, including osteocalcin (SMD 0.70 [0.53, 0.87]) and bone-specific ALP (SMD 0.72 [0.45, 0.99]), while the effect on Procollagen Type 1 N-Terminal Propeptide (P1NP) was directionally favorable but did not reach statistical significance (SMD 0.14 [-0.03, 0.31]) (69). When stratified by agent, liraglutide exhibited the most pronounced anti-resorptive effect [β-CTX SMD -0.41 (-0.59, -0.22)],

Liraglutide also showed significant increases in formation markers, with SMD of 0.57 [0.35, 0.79] for osteocalcin and 0.77 [0.46, 1.08] for bone-specific ALP. The most marked benefits on bone turnover markers were observed in post-menopausal women with T2DM, who exhibited the greatest reduction in β-CTX (SMD -1.43 [-2.01, -0.86]).

A contemporary meta-analysis evaluated the effects of GLP-1RA on multiple bone turnover markers. The assessment of β-CTX showed a significant reduction compared with controls (SMD -0.34, 95% CI -0.54 to -0.14), although moderate heterogeneity was observed. P1NP demonstrated a modest increase (SMD 0.33 [0.07, -0.59]), but findings were inconsistent and heterogeneous, with limited explanatory value from subgroup and meta-regression analyses. In contrast, osteocalcin showed a significant improvement with low heterogeneity, supporting a positive effect on bone formation. For mineral metabolism, a greater increase in 25-OH vitamin D levels with GLP-1RA *versus* controls was reported, with robust results despite heterogeneity. This interaction may run in both directions: in elderly patients with fragility fractures, circulating GLP-1 levels were significantly lower than in age-matched controls and positively correlated with serum vitamin D concentrations (70), although whether vitamin D status modulates incretin secretion, or *vice versa*, has not been established mechanistically. However, evidence for N-terminal-midfragment (N-MID) of osteocalcin was inconclusive due to high variability and lack of robustness. Regarding alkaline phosphatase, total ALP showed no significant changes, while bone-specific ALP increased significantly (MD = 0.91 ug/L, [0.19, 1.63], p=0.01), although this finding was sensitive to the inclusion of a single large study (71). Finally, no significant effects were observed on serum calcium or phosphate levels. Overall, GLP-1RAs appear to modestly reduce bone resorption and enhance selected formation markers, although heterogeneity and variable robustness across outcomes warrant cautious interpretation (16).

Regarding fracture risk, the available evidence remains heterogeneous. A meta-analysis of randomized controlled trials in T2D patients reported 138 fractures among 26,078 participants treated with GLP-1 RAs compared with 154 fractures among 21,745 controls, yielding a pooled relative risk of 0.77 (95% CI: 0.61–0.96) versus other antidiabetic agents or placebo, suggesting a potential fracture-protective effect of the drug class. In details, at a subgroup analysis, the longer duration of the treatment with GLP1-RAs (>78 weeks) showed to be more beneficial on fracture prevention (72). Consistently, a separate meta-analysis found that this protective association became statistically significant only with long-term exposure, i.e. > 52 weeks (RR 0.78, 95% CI 0.65–0.94), corresponding to a number needed to treat of 62 (95% CI 38–156) to prevent one fracture; the authors noted that the modest gains in BMD observed with GLP-1 RAs, although limited in absolute terms, translated into a clinically meaningful reduction in fracture risk when treatment was sustained over time, with an estimated 20% overall reduction in fracture risk that compares favorably with other established preventive strategies in diabetes care (69).

Other independent analyses, however, temper this picture. A 2025 systematic review and meta-analysis of 25 RCTs found no statistically significant reduction in fracture risk with GLP-1 RA use (RR 0.80, 95% CI 0.47–1.36), despite concurrent improvements in BMD and bone turnover markers (BTMs). Nevertheless, comorbidities were largely unreported across the included studies, and BTM measurements are known to be affected by several variables, including age, sex, BMI, physical activity, menstrual status, food intake, and circadian variation. Notably, the included studies did not share consistent characteristics with respect to these variables, a discrepancy that may undermine the homogeneity of the pooled data and limit the overall validity of the conclusions drawn (16). Similarly, a large network meta-analysis of 177 RCTs (n = 165,081) found no evidence that GLP-1 RA-based therapy increased fracture risk relative to insulin, metformin, sulfonylureas, thiazolidinediones, or placebo, concluding overall neutrality rather than protection (73). In contrast, real-world observational data have tended to favor a protective signal: a network meta-analysis of 13 population-based cohort studies (n = 1,064,952) found GLP-1 RA use associated with an approximately 60% reduction in total fracture risk, second only to SGLT2 inhibitors. However, these results should be considered with caution, because of the lack of several important data regarding both diabetic disease, such as duration and severity, and fracture risk, like BMD, BTMs and radiographic confirmation in the most of the included studies (74). Another large Danish nested case–control study (337,648 fracture cases vs 675,296 controls) reported no significant association in unadjusted analysis, but a protective association after adjustment for confounders, while cautioning about possible residual confounding by GLP1-RAs indication (75).

Further complexity arises from the context of diabetes. Although T2D is typically associated with normal or increased BMD, both T1D and T2D confer an elevated fracture risk, reflecting impaired bone quality rather than reduced bone quantity (76–79). Within this framework, GLP-1RA may exert indirect skeletal benefits through improved glycemic control, attenuation of inflammatory pathways, reduction of advanced glycation end products, and enhancement of bone (21). Nonetheless, the overall clinical relevance of these effects on bone health remains to be fully elucidated. A timeline of key clinical evidence on bone outcomes from RCTs, cohort studies and meta-analyses of incretin-based therapies is represented in **Figure 3**.

**Figure 3:**
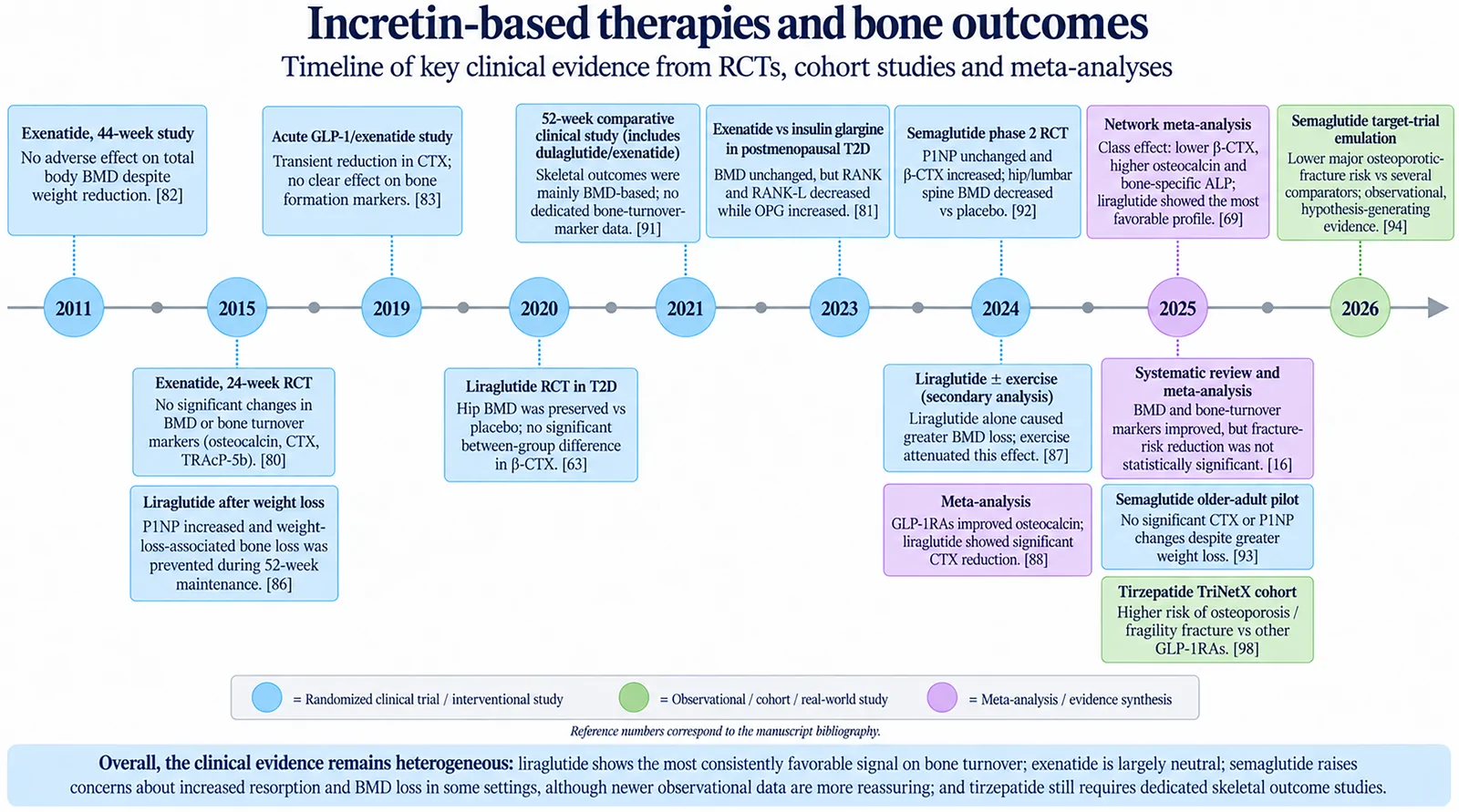
Timeline of key clinical evidence from RCTs, cohort studies and meta-analyses.

### 4.1 Clinical evidence about exenatide

Exenatide is a synthetic exendin-4-based GLP-1 receptor agonist, resistant to DPP-4 degradation, with glucose-dependent insulinotropic effects, glucagon suppression, and delayed gastric emptying; the existence of its short half-life formulation together with the extended-release one determine twice-daily or weekly formulations.

Clinical evidence for exenatide on bone remains limited and overall neutral. In a two-center RCT of 62 patients with newly diagnosed T2D, assigned to exenatide, insulin lispro or pioglitazone for 24 weeks, despite a significant reduction in body weight in the exenatide group (-4.7 ± 0.8 kg), no differences were observed in BMD and bone turnover markers osteocalcin, CTX, and tartrate-resistant acid phosphatase 5b (TRAcP 5b), both compared with baseline or across treatment groups (80).

Similarly, in a 24-week randomized study involving 30 post-menopausal women with T2D naïve to insulin and incretin-based therapies, exenatide was compared with insulin glargine to evaluate its effects on BMD and bone turnover markers. Treatment with exenatide resulted in a significant reduction in body mass index (BMI) compared with glargine, without inducing changes in BMD as assessed by Dual-Energy X-Ray Absorptiometry (DXA). About bone metabolism, no significant differences were observed in conventional biochemical markers of bone turnover, including total ALP, bone-specific ALP, and type 1 crosslinked N-telopeptide, either within or between treatment groups. However, exenatide exerted significant modulatory effects on key regulators of bone remodeling within the OPG/RANK/RANK-L system. Specifically, serum RANK and RANK-L levels were significantly reduced (p=0.009 and p=0.015, respectively), while OPG levels were increased (p=0.02), indicating a shift toward inhibition of osteoclastogenesis and bone resorption. These changes were not observed in the insulin glargine group, in which all measured markers remained stable. These findings suggest that exenatide may exert early anti-resorptive effects through targeted modulation of the OPG/RANK/RANK-L pathway that may not be reflected in the downstream biochemistry (81).

Bunck and colleagues evaluated whether the distinct effects of this treatment on body weight were paralleled by differences in BMD and markers of calcium homeostasis in patients with T2D. Sixty-nine metformin-treated individuals were randomized to receive exenatide twice daily (n=36) or insulin glargine once daily (n=33). Total body BMD and circulating markers of calcium metabolism, were comparable between groups at the end of the study, with both treatments associated with an approximately 6% reduction in body weight. No significant changes were observed in fasting serum alkaline phosphatase, calcium, or phosphate levels. Overall, 44 weeks of treatment with either exenatide or insulin glargine did not adversely affect bone density in patients with T2D, despite their differing impacts on body weight (82).

An acute pharmacokinetic study demonstrated that unlike native GLP-1 (7-36)NH_2_, exenatide, which reached peak plasma concentration at 90 minutes *versus* 15 minutes for native GLP-1, produced a significant reduction of CTX only during the second hour after administration compared with placebo (AUC: -463.1 ± 218 *vs* -136 ± 91% × min; p< 0.05), with no effects on bone formation markers (83).

Regarding the role of exenatide use in modulating fracture risk, a *post-hoc* analysis of the Effects of Once-Weekly Exenatide on Cardiovascular Outcomes in Type 2 Diabetes (EXSCEL) trial (84) addressed this question by examining fracture predictors and the skeletal safety of once-weekly exenatide in a cohort of 14,752 participants followed for a median of 3.2 years. Fractures occurred in only 1.1% of the study population, and incidence did not differ meaningfully between the exenatide and placebo arms (HR 1.11; 95% CI: 0.82-1.51), arguing against a clinically relevant detrimental or protective effect of this agent on bone. Notably, baseline diabetic neuropathy emerged as an independent predictor of fracture, conferring a 50% increase in risk (HR 1.50; 95% CI 1.10–2.05), likely reflecting the contribution of impaired proprioception and balance to fall-related trauma. Given its size and randomized design, this analysis provides some of the most robust evidence available on the skeletal profile of a single GLP-1 RA, though caution is warranted before extending these conclusions to structurally distinct molecules within the class (85).

### 4.2 Clinical evidence about liraglutide

Liraglutide is a long-acting acylated GLP-1 receptor agonist with albumin binding and DPP-4- resistance, with once-daily administration. It is the agent with the largest number of clinical evidence evaluating bone turnover markers, with heterogeneous but more favorable results compared with other GLP-1RA.

In a RCT involving obese non-diabetic women undergoing a hypocaloric diet followed by 52 weeks of weight maintenance with liraglutide 1.2 mg/day, P1NP increased by 16% in the liraglutide group (+7 ± 3 μg/L) compared with a 2% decrease in the control group (-1 ± 4 μg/L; p<0.05), while β-CTX showed no significant changes during the maintenance phase (86). In contrast, in a double-blind RCT including 60 patients with T2D treated with liraglutide 1.8 mg/day or placebo for 26 weeks, β-CTX increased in both groups (liraglutide group +0.07 μg/L; p<0.001 *versus* the placebo group +0.03 μg/L; p=0.04), with no statistically significant difference between groups (p=0.16). P1NP showed an early decline during the first 4 weeks in the liraglutide group, then increased between weeks 4 and 13 and partially recovered; no changes in body weight or P1NP were observed in the placebo treated patients, moreover hip BMD fell in the placebo arm across the study period but stayed stable in the liraglutide arm, a difference between groups that was statistically significant (p = 0.01) (63).

A prespecified secondary analysis of a Danish RCT including 195 obese non-diabetic adults in 4 different arms, treated with liraglutide 3.0 mg/day, moderate-to-vigorous exercise, liraglutide + exercise or placebo group for 52 weeks, following an 800 kcal/day diet, demonstrated that the 8-week hypocaloric phase increased β-CTX by 27% (95% CI 21-35%) and P1NP by 7% (95% CI 4-10%). Subsequently, β-CTX declined from week 26 onward in all groups, with the lowest levels observed in the placebo group, suggesting that high-dose liraglutide does not exert a net anti-resorptive effect beyond placebo in a context of weight loss, while liraglutide treatment alone reduced BMD at clinically relevant sites more than placebo group, and also more than exercise alone despite similar weight loss, while in the liraglutide + exercise group the BMD loss was attenuated (87).

In a 2024 meta-analysis of 26 RCT in T2D patients, liraglutide significantly improved osteocalcin levels (SMD = 2.35; 95% CI: 1.11, 3.60) in subgroup analysis, particularly with treatment durations exceeding 24 weeks, and, as opposed to other GLP-1 RAs, liraglutide has a statistically significant effect in reducing CTX levels (88).

Gilbert’s clinical trial in 2016, conducted over 104 weeks, found that liraglutide monotherapy had no effect on total BMD in patients (89). However, that trial’s dropout rate was as high as 52%, which may have substantially influenced the results. In a meta-analysis conducted by Cheng et al. treatments with liraglutide were associated with a significantly reduced risk of bone fractures compared to placebo and other anti-diabetic drugs (OR, 0.56; 95% CI, 0.38-0.81) (90). Other more recent subgroup analyses confirmed a significant reduction in fracture risk (RR 0.42; 95% CI: 0.21-0.85), while other GLP-1 RAs did not present benefits over other anti-diabetic drug treatments (72).

### 4.3 Clinical evidence about dulaglutide

Dulaglutide is a recombinant fusion protein consisting of a GLP-1 analog linked to a human IgG4 Fc fragment, via a peptide linker, designed to resist DPP-4 enzymatic cleavage and reduce renal clearance. It has a prolonged elimination half-life (~5 days) supporting once-weekly subcutaneous administration, and exerts glucose-dependent insulinotropic activity, glucagon suppression, and delayed gastric emptying through GLP-1 receptor activation.

For dulaglutide, direct evidence on bone turnover markers from clinical studies remains essentially absent. Available literature includes dulaglutide in comparative analyses with other GLP-1RA or insulin, but skeletal outcomes are generally limited to densitometric measurements; specific data on β-CTX, P1NP, osteocalcin, or bone-specific ALP are not reported. A Chinese trial involving 70 patients with T2D treated for 52 weeks with dulaglutide 1.5 mg/week, exenatide 2 mg/week, insulin glargine, or placebo evaluated BMD alone, without biochemical bone markers (91).

In the largest 2024 meta-analysis of 26 RCT in patients with T2D, dulaglutide was mentioned as an included agent; however, results on bone turnover markers were mainly reported as pooled analyses by pharmacological class or in liraglutide-focused subgroup analyses. Dulaglutide was identified as having less pronounced effects than liraglutide on osteocalcin and CTX, although no separate robust estimates were provided for dulaglutide as an individual agent (88).

In summary, no human clinical studies specifically dedicated to bone formation or resorption markers currently exist for dulaglutide, representing a critical evidence gap.

Pooled GLP-1 RA class data suggest a protective trend on fracture risk with treatment beyond 52 weeks (RR 0.80 [0.65-0.94]) (69). However, the isolated dulaglutide estimate within this same network meta-analysis derives from a single trial and carries an extremely wide confidence interval (RR 0.99; 95% CI 0.09-10.78) precluding any clinical interpretation. Similarly, in Zhang Y et al., dulaglutide shows no significant isolated benefit (72). Both sources explicitly note the scarcity of RCTs designed specifically to assess skeletal outcomes with dulaglutide, in contrast to liraglutide’s more mature evidence base.

### 4.4 Clinical evidence about semaglutide

Semaglutide is a long-acting acylated GLP-1 receptor agonist with high albumin binding and DPP-4 resistance, enabling once-weekly subcutaneous or daily oral dosing. It achieves the most potent weight reduction among currently approved GLP-1RA.

Available data on semaglutide from clinical studies specifically focusing on bone turnover markers remain limited but suggest a non-favorable resorption profile in the absence of compensatory effects on bone formation. In a phase 2 double-blind RCT conducted in Denmark involving 64 adults at increased fracture risk randomized to semaglutide 1.0 mg/week or placebo for 52 weeks, semaglutide did not affect P1NP compared (p=0.418) but significantly increased β-CTX relative to placebo (estimated treatment difference: 166.4 ng/L; 95% CI: 25.5, 307.3; p=0.021), drawing up a biochemical profile that promotes bone resorption (92). Hip BMD declined by 2.6% and lumbar spine BMD by 2.1% compared to placebo in the same trial. In a *post hoc* analysis of a pilot trial including 20 older adults with pre-diabetes or T2D treated with semaglutide 1.0 mg/week plus lifestyle counseling for 20 weeks, no significant differences over time were observed in bone turnover markers CTX (p=0.56) or P1NP (p=0.78) between the semaglutide and control groups, despite significantly greater weight loss in the treatment group (-5.3% *vs* -0.89%; p<0.01) (93). The consistently lower BMD and higher bone turnover trends in the semaglutide group of this pilot study underscore the need for larger, adequately powered trials.

In the meta-analysis by Zhang, involving 5 studies with semaglutide initiation, the pooled RR for risk fracture was 0.82 (95% CI: 0.47-1.42), with no beneficial effects (72). Similar results were observed in a meta-analysis by Alalwany, with semaglutide showing favorable fracture trends (RR 0.80; 95%CI 0.46, 1.40) (69). Nevertheless, in a target trial emulation using the TriNetX US Collaborative Network semaglutide initiation was associated with a lower risk of major osteoporotic fracture (MOF) in people with obesity and T2D over three years of follow-up. Compared with empagliflozin, HR was 0.69 (95% CI 0.61-0.77); compared with glipizide, a sulfonylurea, HR 0.72 (0.63-0.83); and compared with usual care, HR 0.84 (0.76-0.93). The comparison versus sitagliptin, a DPP-4i, showed a similar direction (HR 0.84, 0.70-0.99) but lost significance after adjustment. These associations were consistent in per-protocol analyses, site-specific fracture analyses (hip HR range 0.52-0.77 vs. empagliflozin), and a surgical-code-confirmed hip fracture analysis. In contrast, among people with obesity without T2D, no semaglutide comparison reached statistical significance for MOF over two years. The authors interpret the more consistent fracture benefit in the T2D cohort as potentially reflecting a mitigation of hyperglycemia-related bone fragility (via reduced advanced glycation end-product accumulation) that may counterbalance the mechanical unloading expected from GLP-1RA-induced weight loss, though they caution that residual confounding cannot be excluded and describe these findings as hypothesis-generating rather than causal (94).

### 4.5 Clinical evidence about tirzepatide

Tirzepatide is a 39-amino acid synthetic linear peptide and the first approved dual GIP and GLP-1 receptor agonist. C20 fatty diacid-acylation enables reversible albumin binding and DPP-4 resistance, with an about 5-day half-life supporting the subcutaneous administration once a week. Its dual agonism demonstrates superior glycemic efficacy and produces greater weight reduction than selective GLP-1RA, introducing a specific pharmacological tension between the potentially bone-protective effects of GIPr agonism and the bone-resorptive consequences of its more substantial weight loss.

Specific clinical evidence on bone turnover markers with tirzepatide from clinical studies in humans remains limited and indirect. Trends toward a reduction in P1NP and an increase in β-CTX have been noted in patients with T2D after 29 days of tirzepatide treatment in a report from the European Medicines Agency, although the underlying mechanism remains unclear (6). In the Alalwany meta-analysis, only 3 studies with tirzepatide initiation were involved, with non-conclusive fracture outcomes reported (RR 1.13; 95% CI 0.43, 2.92) (69, 95–97).

A retrospective cohort study using the TriNetX database of 66,329 propensity score-matched patients with T2D or obesity who initiated tirzepatide or other GLP-1RA between June 2022 and May 2024 found that tirzepatide was associated with higher risk of new-onset osteoporosis or fragility fracture (HR 1.44; 95% CI: 1.22, 1.69) and initiating osteoporosis therapy (HR 1.61; 95% CI: 1.22, 2.12) relative to other GLP-1RA over a 14-month follow-up (98).

Preclinical data in obese diabetic mice show that tirzepatide promotes bone loss, partially mediated by alterations in gut microbiota composition, with Lachnospiraceae depletion; transplantation of these bacteria ameliorated bone loss through the metabolite evodiamine, which suppresses osteoclastogenesis (99). A separate mouse study comparing semaglutide and tirzepatide in T2D showed divergent bone effects, with distinct impacts on trabecular microarchitecture and turnover markers (6, 100, 101).

These preliminary findings, although far from conclusive, highlight tirzepatide’s complex skeletal pharmacology. The dual GIPr/GLP-1r mechanism may theoretically confer bone-protective advantages, but adequately dedicated studies with pre-specified skeletal endpoints are needed.

### 4.6 Heterogeneity across GLP-1-based agents

The divergent skeletal signatures described above are unlikely to reflect random variability, but rather three converging pharmacological determinants. First, the magnitude of weight loss scales inversely with the net skeletal signal: exenatide and liraglutide, associated with more modest weight reduction (4-6%), show neutral-to-favorable bone turnover profiles, whereas semaglutide and tirzepatide, which produce substantially greater weight loss, show a shift toward resorption (increased β-CTX) that is not offset by a compensatory rise in P1NP. Second, receptor pharmacology differs qualitatively rather than only quantitatively: tirzepatide’s added GIPr agonism is mechanistically anti-resorptive and pro-osteogenic in preclinical models (**Figure 1**), yet this theoretical advantage appears clinically offset by its greater weight-loss potency, indicating that receptor engagement and metabolic consequences act as opposing vectors whose net balance likely depends on dose and treatment duration. Third, methodological heterogeneity across trials, differing in duration (20–52 weeks), population (T2D *vs* obesity, diabetic *vs* non-diabetic), and BTM panel reported, limits direct cross-agent comparison and likely inflates the apparent inconsistency beyond what true biological heterogeneity would predict. In this context, the postmenopausal state contributes significantly to this heterogeneity. The greater response observed in postmenopausal women in some of included studies may partly reflect mechanisms that are not specific to bone tissue. Yang et al. recently showed that women treated with GLP-1RAs achieve higher plasma concentrations at equivalent doses, owing to lower body weight and reduced clearance even at comparable weight, and lose more weight than men, with this difference becoming more pronounced as the overall degree of weight reduction increases. Since weight loss is a known determinant of reduced BMD, through mechanical unloading and changes in marrow adiposity, part of the greater bone response observed in postmenopausal women could be secondary to greater drug exposure and greater weight loss, rather than a direct sex-specific action of incretin-based therapies on bone (102).In addition, many of the studies conducted in humans were retrospective, limiting the ability to infer true cause-and-effect relationships. Moreover, the human studies cited often have small sample sizes or potential misclassification, which may introduce information bias. Finally, there are no existing long-term studies evaluating the use of GLP-1RAs in the treatment of menopause, as most clinical trials only lasted three to six months (103). Our data do not allow these contributions to be disentangled, and future studies stratified by menopausal status, in addition to sex, are needed to clarify this point. Taken together, these observations suggest that the skeletal effect of any incretin-based agent should be interpreted as the net output of a weight-loss-driven resorptive pressure counterbalanced by agent-specific direct receptor signaling, rather than as a fixed, drug-class-level property.

### 4.7 Clinical evidence in type 1 diabetes population

Beyond their established indication for obesity and their well-recognized role in modulating insulin secretion and glycemic control, incretin-based therapies are increasingly finding a place in the management of T1D. GLP-1 RAs and dual GIP/GLP-1 RAs, once considered contraindicated or inappropriate in this population, are now emerging as adjunctive options alongside insulin therapy. This shift has been driven in part by the recent publication of consensus reports (104, 105) providing practical guidelines for the safe use of these agents in people with type 1 diabetes. In parallel, the first studies investigating incretin-based therapies in type 1 diabetes have begun to explore their effects on BTMs or BMD, adding a further dimension to their therapeutic profile in this setting.

Two studies have directly examined incretin-based therapy and skeletal outcomes in T1D, and both point to the same broad conclusion: weight loss achieved with these agents does not appear to translate into measurable bone loss over the studied timeframes. In the randomized, double-blind trial by Johansen et al., 108 adults with T1D on basal-bolus insulin were assigned to thrice-daily exenatide or placebo for 26 weeks; exenatide produced a 4.4 kg greater weight reduction than placebo, yet no between-group differences emerged in BMD at the whole body, hip, lumbar spine, or forearm, nor in the bone turnover markers CTX and P1NP, despite the clinically meaningful weight loss (106). More recently, Al Ozairi et al. reported a real-world observational cohort of 70 people with obesity and T1D treated for 12 months with either a GLP-1RA (70% with liraglutide and 18.6% with semaglutide) or the dual GIP/GLP-1 RA tirzepatide (11.4%); using DXA-derived body composition and bone parameters, the study found clinically meaningful weight loss (-6.33%), reductions in fat and lean mass, and improved HbA1c, while total bone mineral content and BMD remained unchanged overall and even in subgroups stratified by degree of weight loss (107).

These two studies converge on a reassuring signal: neither short-term pharmacological GLP-1RA exposure nor real-world weight loss with GLP-1RA/GIP-GLP-1RA therapy appears to compromise BMD or bone turnover in T1D, even when accompanied by substantial lean mass loss. However, several important limitations constrain the strength of this conclusion. Neither study reports fracture incidence as an outcome, so no direct inference on fragility fracture risk can be drawn, since BMD and BTMs stability do not guarantee unchanged bone strength or microarchitecture. Sample sizes are small (70 patients and considerably fewer per exenatide arm), follow-up is relatively short (26 weeks and 12 months) relative to the usual time course over which bone remodeling and fracture risk typically manifest, and the Al Ozairi study is an uncontrolled, real-world observational design without an untreated comparator group, limiting causal inference and introducing potential confounding [e.g., physical activity, glycemic variability, concomitant Continuous Glucose Monitoring (CGM)/Automated Insulin Delivery (AID) use]. Neither study assessed bone microarchitecture [e.g., trabecular bone score (TBS), High-Resolution peripheral Quantitative Computed Tomography (HR-pQCT)] or performed formal fracture-risk assessment, and heterogeneity in incretin class (exenatide vs. liraglutide/semaglutide/tirzepatide) and mechanism of action (GLP-1 vs. dual GIP/GLP-1 agonism) further limits generalizability across the drug class as a whole.

### 4.8 Clinical evidence in pediatric/adolescent population

In the pediatric and adolescent population (ages 3-18), evidence on skeletal outcomes with incretin-based therapies remains extremely limited, being largely confined to registrational programs [SCALE KIDS for liraglutide (ClinicalTrials.gov ID NCT04775082); STEP TEENS for semaglutide (NCT06571838] and a small number of observational protocols currently registered to monitor bone metabolism. None of these programs has produced robust, longitudinal data on BMD, bone‐turnover markers, or fracture incidence as primary endpoints. The mechanistic plausibility of skeletal effects during this developmental life’s phase is considerable: GLP-1RA-induced weight loss and anorexia during periods of peak bone accrual may compromise the achievement of peak bone mass, a critical determinant of long-term fracture risk (108). Given the mechanistic insights derived from preclinical and adult studies, larger prospective trials and dedicated cohort studies are needed to evaluate the safety and long-term skeletal effects of incretin-based therapies in the pediatric population. Such studies should incorporate pre‐specified skeletal endpoints, adequate pubertal‐stage stratification, and follow‐up long enough to capture bone-accrual trajectories. Particular attention should be directed to potential mediators such as rapid weight loss, nutritional status, and pubertal timing. In the absence of dedicated pediatric guidelines specific to incretin-based therapies, monitoring could reasonably draw on existing ISCD (International Society for Clinical Densitometry) pediatric densitometry standards (109): DXA at the lumbar spine and total body less head, height-adjusted Z-scores, with baseline assessment before treatment initiation and reassessment at 12-month intervals, or every 6 months during phases of rapid weight loss (>0.5–1 SDS BMI decline). Candidate biochemical biomarkers include P1NP and CTX as bone formation/resorption indices and osteocalcin, all interpreted against pediatric pubertal-stage-specific reference ranges rather than adult cut-offs, given their marked physiological fluctuation across puberty (110). Priority research questions include: (i) whether bone-turnover marker trajectories track weight-loss velocity independently of skeletal outcomes; (ii) whether effects differ by pubertal stage at treatment onset; and (iii) whether BMD Z-score changes during incretin-based therapy predict long-term fracture risk into early adulthood. Harmonized monitoring protocols would allow pooled analyses across centers. Until such data are available, use of GLP‐1/GIP receptor agonist in minors should be accompanied by careful skeletal surveillance within clinical studies or registries.

## 5 Conclusions and future perspectives

Incretin hormones constitute a physiologically relevant link between nutrient intake and skeletal remodeling. Both GIP and GLP-1 exert coordinated anabolic and anti-resorptive effects in bone, acting through receptor-mediated signaling in osteoblasts, osteoclasts, osteocytes and MSC, and modulating interconnected pathways including cAMP/PKA, MAPK, Wnt/β-catenin, and RANK-L/OPG. Preclinical evidence is compelling (**Table 1**): disruption of incretin receptor signaling consistently impairs bone mass, microarchitecture, and tissue material properties, while pharmacological activation restores aspects of skeletal integrity. Regarding clinical evidence, findings remain heterogeneous and significantly confounded by weight loss. Among approved GLP-1RA, liraglutide demonstrates the most favorable bone turnover marker profile, while semaglutide raises preliminary concerns for enhanced resorption without compensatory formation. Tirzepatide, with its dual GIPr/GLP-1r mechanism, presents a complex pharmacological profile in which theoretical bone-protective mechanisms may be offset by its more pronounced weight-reducing effects. Data on dulaglutide and exenatide are scarce or neutral, and evidence in the pediatric population is virtually absent (**Table 2**).

**Table 1:** Preclinical and clinical evidence on the skeletal effects of incretin-based therapies.

Preclinical evidences		Clinical evidences	
Cells	Effect	Drugs	Effect
Osteoblasts	↑ cAMP/PKA=↑ ALP, COL1A1, Runx2/Osx; ↑ OPG/↓RANK-L	Exenatide	neutral BTMs early OPG/RANK-l shift only, ↑BMD, =fractures
Osteoclasts	GIP ↓NFATc1/c-FOS/NF-kB = ↓osteoclastogenesis	Liraglutide	↓↑P1NP, ↑osteoclacin, ↓↑β-CTX, ↑ALP, =↓BMD, ↓fractures
Osteocytes	↓ Sclerostin (single study) = Wnt activation	Dulaglutide	No BTM data available, =BMD, non-conclusive data on fractures
MSC	↑ osteogenic *vs* ↓ adipogenic commitment (MAPk, Wnt)	Semaglutide	↑ β-CTX, ↓hip and spine BMD, =↓fractures
Genetic Mouse Models	GIPr-KO/GLP-1r-KO/DIRKO: ↓BV7TV, ↓strength, ↓matrix quality	Tirzepatide	Trend ↑β-CTX; ↑fracture/osteoporosis risk

**Table 2:** Clinical evidence of incretin-based therapies on bone turn-over markers.

Drug	Effects on bone resorption	Effects on bone formation	References
Exenatide	= CTX ↑ OPG/RANK-L = TRAcP 5	= osteocalcin = ALP	(80–83)
Liraglutide	↓↑ β-CTX	↑ osteocalcin ↑ ALP ↓↑ P1NP	(63, 69, 86–88)
Dulaglutide	No evidence	No evidence	
Semaglutide	↑= CTX	= P1NP	(92, 93)
Tirzepatide	No evidence	No evidence	

CTX, C-terminal Telopeptide of Type I Collagen; OPG, Osteoprotegerin; RANK-L, Receptor Activator of Nuclear Factor Kappa-B Ligand; TRAcP 5, Tartrate-Resistant Acid Phosphatase 5b; ALP, Alkaline Phosphatase; P1NP, Procollagen Type I N-Terminal Propeptide.

Several critical priorities emerge for future research. First, prospective trials specifically designed with bone endpoints are needed for all incretin-based agents and are essentially mandatory for tirzepatide and the emerging triple agonists. Second, mechanistic studies capable of dissociating the direct receptor-mediated skeletal effects of incretins from the indirect consequences of weight loss and altered nutritional intake are fundamental to interpreting clinical data. To this end, future studies should incorporate a methodological framework - such as logistic regression models adjusted for changes in body weight, BMI, or waist circumference - to determine whether the observed variations in skeletal outcomes are attributable to incretin-specific mechanisms rather than to weight loss itself. Third, the potential for exercise, nutritional supplementation or co-administration of bone-protective agents to mitigate GLP-1RA-related skeletal risks warrants systematic evaluation. Fourth, the pediatric and adolescent population requires urgent dedicated longitudinal study. Finally, the bidirectional interaction between bone-derived osteocalcin and incretin secretion from intestinal L-cells positions the skeleton as an active participant in the regulation of energy metabolism, a conceptual framework with potential therapeutic implications. Clarifying whether and how the incretin-bone axis can be selectively harnessed to preserve skeletal integrity during metabolic disease management represents one of the most clinically relevant challenges at the intersection of endocrinology and bone biology.
